# The treatment of diabetic macular edema with intravitreal bevacizumab in users of the Brazilian public health system

**DOI:** 10.5935/0004-2749.2024-0146

**Published:** 2024-08-30

**Authors:** Larissa Fouad Ibrahim, Aleida Nazareth Soares, Lucas Assis Costa, Thabata Machado Correia Domingues, Alessandra Hubner de Souza

**Affiliations:** 1 Faculdade Ciências Médicas de Minas Gerais Belo Horizonte Minas Gerais Brazil Faculdade Ciências Médicas de Minas Gerais, Belo Horizonte, Minas Gerais, Brazil; 2 Instituto de Olhos Ciências Médicas de Minas Gerais Belo Horizonte Minas Gerais Brazil Instituto de Olhos Ciências Médicas de Minas Gerais, Belo Horizonte, Minas Gerais, Brazil

Dear editor,

Diabetic macular edema (DME) is the primary contributor to reduced visual acuity (VA) in people with diabetes mellitus^(1)^. The multiple treatment options include the angiogenic drug, bevacizumab, a vascular endothelial growth inhibitor. Treatment of DME is an off-label use of bevacizumab but, due to its good treatment results and low cost, it is the therapy of choice in many public health systems^(2)^. This study aimed to evaluate DME treatment with antiangiogenic drugs, in terms of both visual and economic outcomes within the Brazilian public health system (SUS). It is hoped that our findings will contribute to the development of eviden cebased public health policies.

The participants in this single-center retrospective study were adults diagnosed with DME and indicated for intravitreal bevacizumab therapy at the Instituto de Olhos, Ciências Médicas, in Belo Horizonte, Minas Gerais, Brazil. Data were collected from the period between January 2018, and March 2023.

The inclusion criterion was at least 12 months of follow-up. Patients with uveitis, uncontrolled glaucoma, vitreous hemorrhage, vitreomacular traction, or any evidence of macular fibrovascular proliferation or media opacity that could affect VA and the quality of retinal images were excluded. All the patients were treated with bevacizumab (1.25 mg / 0.05 ml) on a pro re nata (as needed) basis. Data were obtained from the institution’s medical records, including each patient’s optical coherence tomography (OCT) scan results.

The outcome variables were the mean changes in best corrected visual acuity (BCVA) in logMAR at 1,3,6 and 12 months of follow up, changes in central macular thickness (CMT) measurements (in µm) obtained by OCT at 12 months, in relation to baseline measurements, and the cost to treat each eye in reais (R$). A secondary outcome variable was the number of injections administered during the follow up period.

A total of 130 eyes treated for DME with intravitreal bevacizumab were followed up for 1 year. The mean age of participants was 63.3 (±9.5) years, with a range of 39-85 years 53% of the participants were men and 47% were women.

Over 12 months, 62% of the patients received three injections, 25.6% received 4-6 injections, 8.5% received 1-2 injections, and 3.8% received 7-9 injections. As can be seen in Table 1, 63.84% of the participants showed improvements in their BCVA (≥ one line in ETDRS chart)^(3)^ after 12 months. Only 12.31% showed worsening of their BCVA (≥ one line). The remaining 23.85% maintained their original BCVA after 1 year.

**Table 1:** Changes in visual acuity over time compared to baseline in patients with diabetic macular edema treated with intravitreal bevacizumab (measured with ETDRS chart)

Visual acuity results	After 1 month (n=119)	After 3 months (n=122)	After 6 months (n=112)	After 12 months (n=130)
	n (%)			
Improvement in BCVA compared to baseline				
1 line	29 (24.37)	27 (22.13)	26 (23.21)	25 (19.23)
2 lines	21 (17.65)	23 (18.85)	22 (19.64)	27 (20.77)
3 lines	7 (5.88)	14 (11.48)	14 (12.50)	21 (16.15)
≥4 lines	3 (2.52)	6 (4.92)	7 (6.25)	10 (7.69)
Worsening of BCVA compared to baseline				
1 line	12 (10.08)	11 (9.02)	12 (10.71)	9 (6.92)
2 lines	6 (5.04)	6 (4.92)	3 (2.68)	6 (4.62)
3 lines	0 (0.00)	0 (0.00)	3 (2.68)	1 (0.77)
≥4 lines	1 (0.84)	0 (0.00)	0 (0.00)	0 (0.00)

BCVA= best corrected visual acuity.

Between baseline and 12 months, there was a median reduction in participant CMT of 39.5 µm.

Calculation of the treatment costs over 1 year gave a total of R$92,577.02 for all 130 eyes. The estimated expenditure per eye was R$711.98.

Overall, this study showed positive clinical results from bevacizumab treatment of DME. After a year, an improvement in VA of at least one line was observed in 63.8% of participants, with a gain of two lines or more in 44.62%. These results are comparable to those seen in previous studies^(4)^, but are lower than those obtained by the Diabetic Retinopathy Clinical Research Network using protocol T, which achieved an average gain of 9.7 letters (equivalent to 2 lines) in participants^(5)^. The lower level of VA improvement in our participants may be attributable to their receipt of fewer injections (mean injections per year were 3 in our participants vs. 10 in protocol T participants). However, further follow-up of our participants to monitor more long-term improvements in VA can be difficult as patients become less inclined to continue with regular return visits if they experience no further problems.

Our results suggest that bevacizumab is a feasible treatment for DME, particularly in services with high economic demands. In practical terms, the fiscal impact of DME on the SUS is considerable, due to a high prevalence of patients receiving treatment for the condition. A previous study evaluated the budgetary impact on the Minas Gerais State Health Department of SUS treatment of DME. The study compared three medications commonly used to treat DME (bevacizumab, aflibercept, and ranibizumab) over 5 years. The budgetary impact, accounting for measured demands and estimates of epidemiological demands, was R$69,493,906.95-473,226,278.78 for bevacizumab; R$349,319,965.60-2,378,732,103.09 for ranibizumab; and R$543,867,485.47-3,703,524,490.16 for aflibercept. Bevacizumab was the most cost-effective and viable alternative in all estimate scenarios and sensitivity analyses^(4)^.

In patients treated with bevacizumab for DME, the visual outcomes obtained could be improved by identifying ways to increase injection frequency, optimize follow-up, and control the underlying disease. Indications for intravitreal bevacizumab therapy should be assessed individually in each patient, taking their likely visual prognosis into account to further optimize the cost-effectiveness of this treatment option.
